# Call to hospital times for suspected stroke patients in the North East of England: a service evaluation

**DOI:** 10.29045/14784726.2019.09.4.2.31

**Published:** 2019-09-01

**Authors:** Daniel Haworth, Graham McClelland

**Affiliations:** North East Ambulance Service NHS Foundation Trust: ORCID iD: https://orcid.org/0000-0003-0334-3300; North East Ambulance Service NHS Foundation Trust: ORCID iD: https://orcid.org/0000-0002-4502-5821

**Keywords:** service evaluation, stroke, timings

## Abstract

**Introduction:**

Stroke is a leading cause of mortality and morbidity. The role of the ambulance service in acute stroke care focuses on recognition followed by rapid transport to specialist care. The treatment options for acute ischaemic strokes are time dependent, so minimising the pre-hospital phase of care is important. The aim of this service evaluation was to report historical pre-hospital times for suspected stroke patients transported by the North East Ambulance Service NHS Foundation Trust (NEAS) and identify areas for improvement.

**Methods:**

This was a retrospective service evaluation using routinely collected data. Data on overall call to hospital times, call to arrival times, on scene times and leave scene to hospital are reported.

**Results:**

Data on 24,070 patients with an impression of stroke transported by NEAS between 1 April 2011 and 31 May 2018 are reported. The median call to hospital time increased from 41 to 68 minutes, call to arrival from 7 to 17 minutes, on scene from 20 to 30 minutes and leave to hospital from 12 to 15 minutes.

**Conclusion:**

The pre-hospital call to hospital time for stroke patients increased between 2011 and 2018. The call to arrival phase saw a sharp increase between 2015 and 2017, whereas on scene and leave scene to hospital saw steadier increases. Increasing demand on the ambulance service, reorganisation of regional stroke services and other factors may have contributed to the increase in times. Reducing the on scene phase of pre-hospital stroke care would lead to patient benefits and is the area where ambulance clinicians have the most influence.

## Introduction

Stroke is the fourth leading cause of death in the UK and a leading cause of long-term disability (Stroke Association, 2018). Acute stroke services are structured around hyper acute stroke units (HASUs) that provide centralised, specialist care and have been shown to improve patient outcomes (Morris et al., 2014). Centralisation offers faster access to multidisciplinary teams providing specialist care, neuroimaging and reperfusion therapy, but potentially at the expense of prolonged pre-hospital times. Rapid access to specialist stroke care is important for all stroke patients. Rapid assessment by stroke specialists is particularly pertinent for acute ischaemic stroke patients as the reperfusion treatment options, thrombolysis and mechanical thrombectomy, have short therapeutic windows and the benefits are time dependent (Emberson et al., 2014; Evans, White, Cowley, & Werring, 2017).

Within the UK, regional ambulance services provide the initial response to medical emergencies and are the first point of contact with the healthcare system for the majority of acute stroke patients (Price et al., 2013). Rapid identification of suspected stroke patients and transport to specialist care are the foci of the ambulance service in acute stroke care. This is because there are no reliable methods of differentiating stroke aetiologies, or deliverable treatments, in normal pre-hospital practice. One of the actions that ambulance clinicians should undertake is pre-notification to alert the receiving stroke unit, as this has been shown to reduce the time to critical interventions in hospital, which should improve patient outcomes (Sheppard et al., 2015).

Due to the time dependent nature of the treatment options, currently 4.5 hours from onset for thrombolysis, and the lack of meaningful interventions that can be performed in the pre-hospital setting, minimising the pre-hospital time is important. National guidelines (Association of Ambulance Chief Executives, 2016; Intercollegiate Stroke Working Party, 2016; NICE, 2017) all recommend rapid access to specialist stroke services for suspected stroke patients identified using validated tools such as the Face Arms Speech Test (FAST) (Harbison et al., 2003) and minimising the time spent in the pre-hospital setting.

The North East Ambulance Service NHS Foundation Trust (NEAS) was a high performing service in terms of stroke care according to the national ambulance quality indicators (AQIs) (NHS England, 2019a). NEAS was consistently ranked first or second until 2014. Since 2014 there has been a significant deterioration in NEAS performance against the stroke AQI.

The AQI for stroke changed in 2017. Prior to 2017 ambulance services reported the percentage of stroke patients arriving at a HASU within 60 minutes and the compliance with the stroke care bundle. The stroke care bundle included recording of the FAST, blood sugar and blood pressure. NEAS, and the other ambulance trusts, all consistently scored > 95% on care bundle compliance, whereas the percentage of patients transported to a HASU varied. The current AQI reports the mean call to hospital time, the 50th and 90th centile and timings extracted from the Sentinel Stroke National Audit Programme (SSNAP) related to stroke patients admitted by ambulance.

Within the NEAS area of operations there has been significant reorganisation of services, including stroke, over recent years. There were 10 hospitals receiving stroke patients in 2011 which centralised to six hospitals by 2018. The six hospitals in 2018 had four different pathways for NEAS to access with stroke patients. These are described as: direct to computed tomography (CT); direct to the emergency department (ED); direct to stroke ward; or a hybrid approach.

This service evaluation sought to report any changes in the pre-hospital times for stroke patients and identify areas for improvement.

## Methods

This study was a retrospective service evaluation conducted using routinely collected data.

### Setting

NEAS is the regional ambulance provider for around 2.5 million people in North East England covering Northumberland, Tyne and Wear, County Durham, Darlington and Teesside. NEAS employs around 1200 front line clinicians (paramedics and other clinical roles) who work out of 56 stations across the region.

Anecdotal reports within NEAS suggest that how the pre-hospital clinician interacts with the hospital affects the on scene time; therefore the four differing pathways used by the six regional HASUs were explored to see if they had any impact on the pre-hospital on scene times.

### Data collection and analysis

Timing data on all patients with a clinician impression of stroke recorded between 1 April 2011 and 31 May 2018 were requested from the NEAS informatics team. Clinician impression of stroke is primarily based upon FAST but also includes non-FAST suspected stroke. Data were extracted from the NEAS contact centre data linked with ambulance clinician impression. These data represent patients with a suspected stroke diagnosis who are referred to as stroke patients from this point on. Patient records with no evidence of transport to hospital were removed.

All data are presented in a descriptive fashion. All times are presented as median values with interquartile ranges (IQRs).

## Results

**Figure 1:**
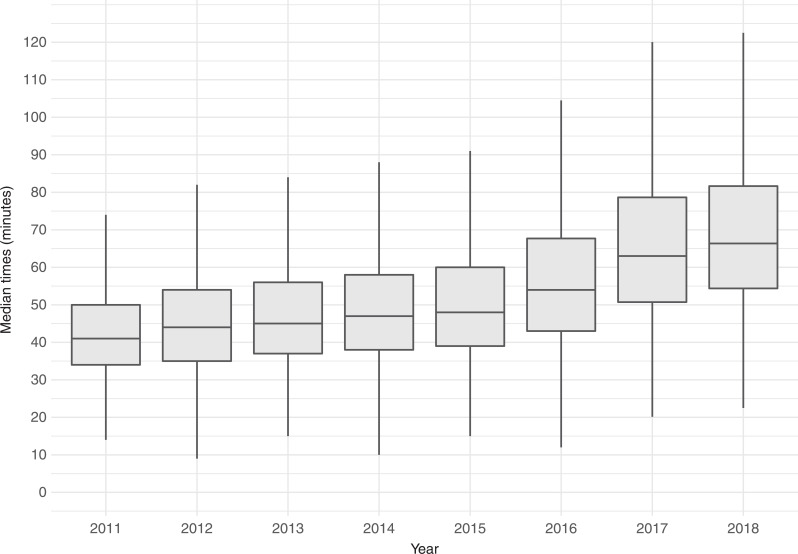
Figure 1. Boxplot of call to hospital times.

The data on 24,070 stroke patients transported by NEAS are summarised in Table 1. Individual timing phases are shown in Table 1 and Figure 1.

**Table 1.** Data on stroke patients transported by the North East Ambulance Service NHS Foundation Trust from 2011 to 2018.

Year	Patients	Call to hospital (IQR)	Call to arrive (IQR)	Arrive to leave (IQR)	Leave to hospital (IQR)
2011*	1010	41 (34–50)	7 (5–11)	20 (16–26)	12 (7–17)
2012	3317	44 (35–54)	7 (5–11)	22 (17–28)	13 (8–19)
2013	3327	45 (37–56)	7 (5–11)	23 (18–29)	13 (8–19)
2014	3604	47 (38–59)	8 (5–13)	24 (18–31)	13 (8–19)
2015	3549	48 (39–60)	8 (5–12)	25 (19–32)	13 (9–19)
2016	3497	55 (43–69)	10 (6–17)	26 (20–34)	14 (9–20)
2017	4063	64 (51–83)	15 (9–27)	28 (21–38)	14 (10–21)
2018**	1703	68 (55–86)	17 (10–30)	30 (22–39)	15 (10–21)

Note: Times are reported as median number of minutes. IQR = interquartile range. *2011 is based on nine months’ data; **2018 is based on five months’ data.

The median overall times from NEAS receiving the call to the patient arriving at the hospital are shown in Figure 1.

**Figure 2:**
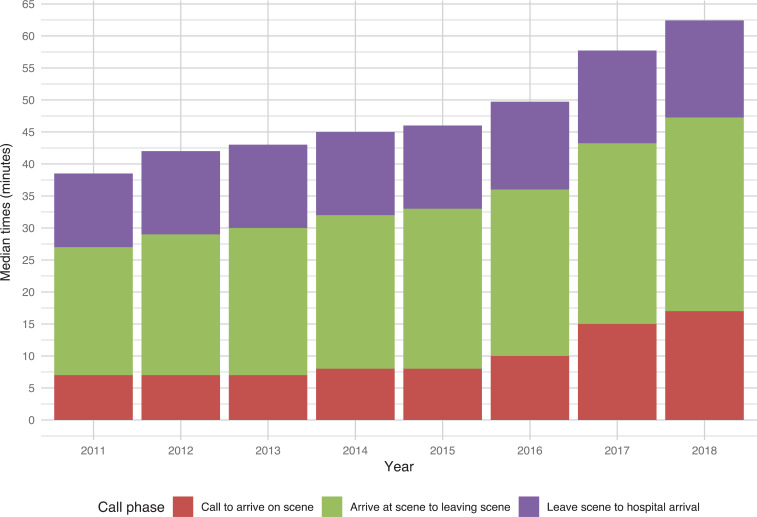
Figure 2. Call to hospital times including constituent phases.

The median times for the three individual phases (call to arrive, arrive to leave, leave to hospital) that comprise the call to hospital time show an increase in median time for all phases of the emergency call (Figure 2).

**Figure 3:**
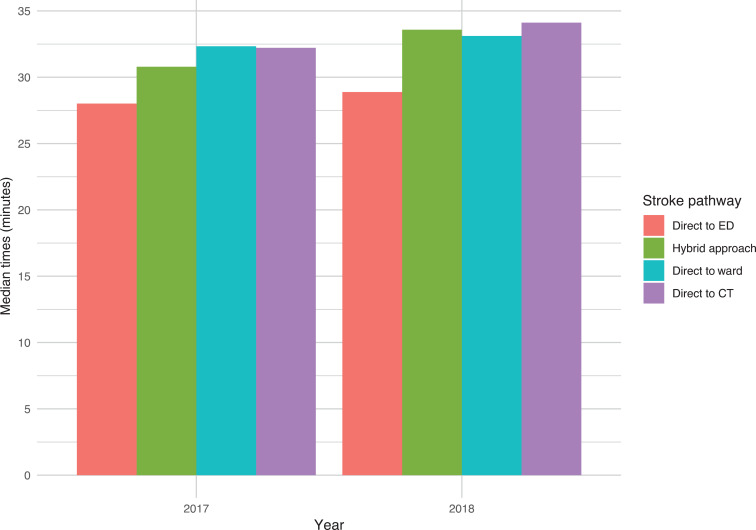
Figure 3. On scene time based on available stroke pathway.

Note: CT = computed tomography; ED = emergency department.

The differing hospital access pathways (direct to CT (one hospital), ED (two hospitals), stroke ward (one hospital) or hybrid (two hospitals)) are shown in Figure 3, to display the impact on the on scene time.

## Discussion

Over the seven years reported there has been a steady increase in times across all the pre-hospital phases of stroke care. This is an unwanted finding in light of calls to streamline the pre-hospital phase of acute stroke management (Fassbender et al., 2013). Each individual time section increased – call to arrival from 7 to 17 (243%) minutes, on scene from 20 to 30 (150%) minutes and leave to hospital from 12 to 15 (125%) minutes. The median call to hospital time, encompassing the whole patient encounter, increased from 41 to 68 (166%) minutes.

The steady increase in stroke timings, reflected in deteriorating NEAS performance against the stroke AQI between 2011 and 2017, mirrors a steady decline against all time-based performance targets, presumably due to a general increase in demand and pressure on the service.

A sharp rise was seen in the call to arrival times between 2015 and 2017 with no obvious explanation. Call to arrival times need to be examined with caution as this evaluation is based on clinician impression of stroke so it is unknown how many of these calls were categorised as stroke by the dispatchers. However, one factor that may be relevant to the increase in pre-hospital stroke timings is that in 2017 NEAS implemented the Ambulance Response Programme (ARP) (NHS England, 2019b). The ARP changed how all calls to the ambulance service were categorised and how resources were dispatched. It specifically impacted on stroke care by changing both the categorisation and the resources dispatched. Stroke was changed from a red call requiring an 8-minute response to a category two call requiring an average response within 18 minutes. The ARP also changed the response model, in that it required a vehicle capable of transporting the patient (a double crewed ambulance as opposed to a response car) to be dispatched. Prior to the ARP a response car could be the initial response which then had to wait for a transporting vehicle. The overall impact of these changes should be a shorter overall call to hospital time where the extended response time is offset by a reduced on scene time. The ARP was intended to improve the whole acute stroke system performance, which includes pre-hospital and hospital-based elements, so it is difficult to look at the pre-hospital data in isolation. However, the ARP was only implemented in October 2017 so other factors, such as the increasing pressure on the whole of NEAS, will have contributed to the increase in call to arrival times.

The travel times, call to arrival and leave to hospital, may also have been affected by the centralisation of stroke services within the NEAS area of operations. Over the time period described, the number of hospitals receiving stroke patients reduced by 40% and the number of stroke patients transported by NEAS increased by 22% (based on 2012 to 2017). This centralisation increased the distance that ambulance crews travelled, and therefore will have increased the times. The travel times are largely dictated by consistent external factors such as geography, weather, road networks and service pressures, whereas the on scene times are more dependent on the patient presentation and the ambulance crew behaviour.

On scene times steadily increased across the study despite no discernible change in pre-hospital care for stroke patients. Li et al. (2018) reported on barriers to pre-hospital care and the impact that these had on the on scene times. They identified five categories of barrier: access; assessment and management; communication; extrication and transport; and refusal. Access, communication and extrication were common barriers and the presence of these was associated with extended on scene times. Without further research into what factors were and are encountered by NEAS clinicians, it is difficult to identify whether there are specific local issues and what can be done to address these. In Helsinki, Finland, a simple training programme reduced the on scene time for stroke patients attended by emergency medical services (EMS) from 25 minutes to 22.5 minutes, showing that improvement is possible (Puolakka et al., 2016).

Other studies have reported pre-hospital times from various settings. The London Ambulance Service reported a median response time (call to arrival) of 7 minutes, a median on scene time of 31 minutes and a median leave to hospital time of 15 minutes in 2016/2017 (London Ambulance Service, 2017). Schwartz, Dreyer, Murugiah, and Ranasinghe (2016) described a large cohort of patients with an impression of stroke transported by EMS in the USA. The median call to arrival (call centre + dispatch + time to scene) was 7 minutes, the on scene time was 15 minutes and the transport time was 12 minutes. Mosley et al. (2007) reported on pre-hospital timelines for stroke patients transported by EMS in Melbourne, Australia and reported a median response time of 12 (IQR, 9–18) minutes, on scene time of 16 (IQR, 12–20) minutes, transport time of 15 (IQR, 10–20) minutes and total ambulance service time of 44 (IQR, 37–54) minutes. Drenck et al. (2019) reported median on scene times of 21 minutes (IQR, 16–27) in Denmark. Compared to these studies the recent (2017 and 2018) NEAS call to scene and on scene times appear extended.

The variety of hospital access routes in the North East reflects variability in many aspects of pre-hospital stroke care which can be seen at a national level (McClelland, Rodgers, & Price, 2018). Although the direct to ED route minimises the on scene time and therefore appears the preferable route from a pre-hospital standpoint, this needs to be taken in context of the whole patient journey. Other routes, such as direct to stroke ward and direct to CT, may reduce the time to specialist care and critical interventions in hospital which directly impact on patient outcomes. Without examining data on the whole patient journey from initial call to the ambulance service through to hospital discharge, it is impossible to say which route produces the best patient outcome. Pre-hospital data should be added to the SSNAP in 2019, which will allow these analyses to be undertaken.

Based on the thinking that time is brain and that minutes count in acute stroke care (Saver, 2006), then minimising the pre-hospital time is desirable. Pulvers and Watson (2017) reviewed studies reporting timelines in acute stroke and described little change over two decades. They also stated that ambulance services were key to rapid transport to hospital which then improves patient outcomes via faster assessment, diagnosis and treatment. The data presented from this service evaluation show that there has been a steady deterioration in pre-hospital stroke times in the North East between 2011 and 2018. Improvements in travel times (call to arrival and leave to hospital) are likely to be difficult due to the influence of external factors. Improvements should be possible in on scene times, acknowledging the barriers identified by Li et al. (2018), as this is less influenced by external factors and within the power of ambulance clinicians to influence.

## Conclusion

Pre-hospital stroke times have increased between 2011 and 2018 despite reductions being desirable. A large part of the increase in overall times is due to the call to arrival phase which may have been influenced by pressures across the whole ambulance service. Travel times from the scene to the hospital have increased but this probably reflects wider service pressures and centralisation of specialist stroke services. On scene times have seen a steady increase and account for the largest individual phase of time. On scene times may be the area that NEAS has the greatest ability to influence in order to reduce the overall call to hospital time and improve patient outcomes.

## Author contributions

DH conducted the initial service evaluation. DH and GM jointly interpreted the data, and conceived, drafted and approved the manuscript.

## Conflict of interest

None declared.

## Ethics

Not required.

## Funding

None.
